# Preprocedural 3D Transesophageal Echocardiography for the Prediction of Device Deformation Morphology and Peri-Device Leaks After Transcatheter Left Atrial Appendage Occlusion with the Amplatzer^TM^ Device

**DOI:** 10.3390/jcm14124211

**Published:** 2025-06-13

**Authors:** Annemarie Kirschfink, Andreas Puetz, Michael Frick, Rami Al-Khusein, Pieterjan van Rijckeghem, Anas Alnaimi, Kinan Kneizeh, Felix Vogt, Nikolaus Marx, Ertunc Altiok, Jörg Schroeder

**Affiliations:** Department of Cardiology, Angiology and Intensive Care, University Hospital, RWTH Aachen University, Pauwelsstrasse 30, 52074 Aachen, Germany

**Keywords:** left atrial appendage occlusion, Amplatzer^TM^ device, transesophageal echocardiography, conventional cardiac angiography, peri-device leak

## Abstract

**Background/Objectives**: Percutaneous left atrial appendage occlusion (LAAO) has become an alternative to oral anticoagulation in selected patients with atrial fibrillation. The results of transcatheter LAAO were evaluated by conventional cardiac angiography (CCA), fluoroscopy, and 2D and 3D transesophageal echocardiography (TEE). **Methods**: In 47 consecutive patients (76 ± 8 years), LAAO was performed with the Amplatzer^TM^ device. CCA and 2D and 3D TEE were performed for LAA measurements. The eccentricity of the device landing zone was assessed by 3D TEE as the ratio of the maximal and minimal diameters. The device size was selected depending on the CCA maximal diameter. The postprocedural device lobe morphology was classified by fluoroscopy as “square” type (sign of undersizing), “tire” type (optimal deformation), and “strawberry” type (sign of oversizing). After 6 months, TEE was repeated to evaluate peri-device leaks (PDLs). **Results**: The postprocedural device morphology was “square” type in 9 (19%), “tire” type in 28 (60%), and “strawberry” type in 10 patients (21%). After 6 months, peri-device leaks were observed in 11 patients (23%), with the lowest incidence in the “tire”-type group (11%) compared with “square” type (56%) and “strawberry” type (30%) (*p* = 0.019). The 3D TEE eccentricity index with a cut-off value of ≤1.29 (indicating a more circular morphology) was a very specific predictor for excluding postprocedural device deformation of the “strawberry” type (AUC = 0.689; sensitivity 48.7%, specificity 100%). **Conclusions**: Undersizing as well as oversizing of the LAA occluder, as characterized by deformation type by fluoroscopy, was associated with postprocedural PDLs. The eccentricity of the LAA landing zone by 3D TEE may indicate inadequate size selection of the Amplatzer^TM^ device leading to oversizing in patients with a more eccentric LAA.

## 1. Introduction

Atrial fibrillation is the most common arrhythmia in adults and causes an increased risk of ischemic stroke [1]. Percutaneous occlusion of the left atrial appendage (LAA) has become an alternative to oral anticoagulation in selected patients with nonvalvular atrial fibrillation and may be considered in patients in whom oral anticoagulation is contraindicated according to the current American and European guidelines [2,3]. Besides surgical approaches with occlusion or exclusion of the LAA, different transcatheter devices have been developed for LAA occlusion (LAAO): the most frequently used are the plug-type WATCHMAN^TM^ device (Boston Scientific, Marlborough, MA, USA) and the pacifier principle Amplatzer^TM^ device (Abbott Vascular, Santa Clara, CA, USA). In contrast to the single-occlusive WATCHMAN^TM^ device, both Amplatzer^TM^ devices are dual-occlusive and consist of a lobe and a disc [4].

LAA size and anatomy are highly variable [5]. Therefore, a transesophageal echocardiography (TEE) is recommended for the selection of the right device size [6].

During LAA occluder implantation, two different imaging modalities should be used for device size selection according to the Amplatzer^TM^ instructions for use. Common imaging modalities that are applied in LAAO are 2D TEE and conventional cardiac angiography (CCA). Computed tomography can also be used as an alternative tool. Several studies have shown that 2D TEE diameters tend to be smaller than those measured by cardiac computed tomography. CT also seems to be superior in the determination of the correct size of the device [7,8,9]. Incorrect sizing measurements may lead to unfavorable results and complications like rare late device migration or peri-device leaks (PDLs), which are common [4,6]. The amount of eccentricity of the LAA ostium has also been proposed to predict PDLs. However, there is conflicting evidence on whether the eccentricity of the LAA or the mis-sizing of the device leads to this phenomenon [10,11,12]. In summary, TEE and CCA imaging are primarily used to measure the diameter of the LAA landing zone to select the appropriate LAAO device size in clinical practice [9,13].

The purpose of this study was to compare 2D TEE and 3D TEE measurements with conventional cardiac angiography (CCA) and fluoroscopy in LAAO with the Amplatzer^TM^ device and their ability to assess the eccentricity of the LAA ostium as well as their impact on unfavorable results.

## 2. Materials and Methods

In this single-center, retrospective study, 47 consecutive patients with atrial fibrillation who underwent LAAO with the Amplatzer^TM^ device (16 (34%) Amplatzer^TM^ Cardiac Plug (ACP); 31 (66%) Amulet^TM^) were included. This study was approved by the local ethics committee of the faculty of medicine at the RWTH Aachen University, and the need for consent was waived (EK 24-104). This study was conducted in accordance with the guidelines of the Declaration of Helsinki.

### 2.1. TEE Protocol

A preprocedural TEE was performed in all the patients in order to exclude unfavorable anatomy of LAA or LAA thrombus. Preprocedural TEE was conducted within 48 h prior to intervention in the left lateral position in patients fasting for at least 6 h. Mild sedation was induced by 1–4 mg of midazolam. Two-dimensional TEE images of the LAA were obtained at 45°, 90°, and 135°. LAA visibility at 0° was not sufficient in about 15% of patients, and therefore, this view was not included in the further analysis. Moreover, 3D zoom images of the LAA were acquired. An experienced cardiologist in interventional imaging performed intraprocedural TEE under general anesthesia. Furthermore, 2D and 3D TEE images were obtained in order to guide the LAA occluder implantation following the current guidelines, to exclude acute PDLs and pericardial effusion, and to evaluate device position and stability [6,14]. At the 6-month follow-up, 2D and 3D TEE without and with color Doppler were repeated to evaluate the presence of device-related thrombi and PDLs of any severity.

The 2D and 3D TEE images were obtained by commercially available echocardiographic systems (EPIQ 7c; Release 9.0.14; Philips Medical Systems, Andover, MA, USA) with a 3D transesophageal probe (X7-2t; Philips Medical Systems, Andover, MA, USA). Dedicated software (QLAB; Release 8.0; Philips Medical Systems, Andover, MA, USA) was used for off-line analysis of the 3D data.

### 2.2. Imaging for LAA Sizing

#### 2.2.1. Measurement by TEE

Preprocedural and intraprocedural 2D TEE images of the LAA were obtained at 45°, 90°, and 135°, and the maximal width of the LAA ostium and the width of the landing zone at the level of the left circumflex artery were measured [6,14].

Additionally, 3D zoom images of the LAA were obtained for measuring the area and the maximal and minimal diameters of the device lobe landing zone. A cross-sectional area within the 3D image was established approximately 10 mm distal to the LAA ostium. Here, the maximal and minimal diameters were accessed. Moreover, an area (*A*)-derived mean diameter (*d*) was calculated as $d=2\times \sqrt{\frac{A}{\pi }}$ as well as an eccentricity index as a ratio of the maximal and minimal 3D diameters for assessing the eccentricity of the device landing zone of the LAA.

#### 2.2.2. Measurement by Angiography

**Figure 1:**
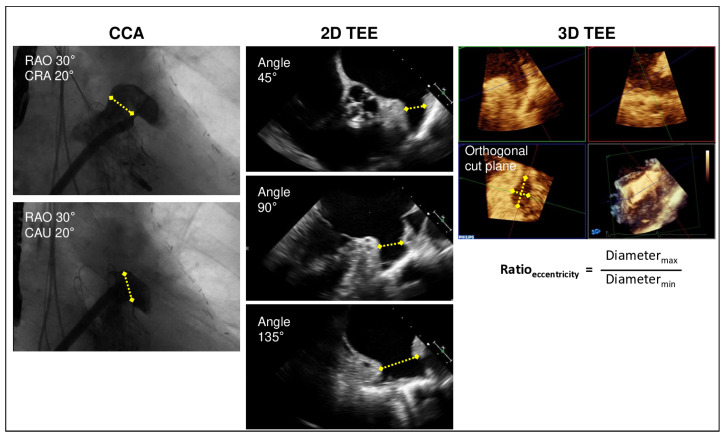
Sizing of LAA landing zone. Measurements of diameters (yellow arrows) by conventional cardiac angiography (CCA) in two projections: by 2D transesophageal echocardiography (TEE) in 45°, 90°, and 135° views as well as 3D TEE cut-plane view of the LAA landing zone and calculation of 3D eccentricity index as the ratio of the maximal and minimal diameters.

Before LAA occluder implantation, an intraprocedural LAA angiography was performed to measure the maximal LAA diameters of the landing zone (10 mm beneath the LA ostium) in two projections (right anterior oblique (RAO) 30° cranial 20 and RAO 30° caudal 20°) (Figure 1). The LAA occluder size was selected according to the measurement by angiography.

### 2.3. LAA Occluder Implantation

In all the procedures, an Amplatzer^TM^ device was implanted by experienced interventional operators who were advised by a device-specific proctor following the device’s instructions for use. Transcatheter LAA occluder implantation was successfully performed under general anesthesia in all the patients. For intraprocedural guidance, CCA and fluoroscopy as well as 2D and 3D TEE were used as described above.

**Figure 2:**
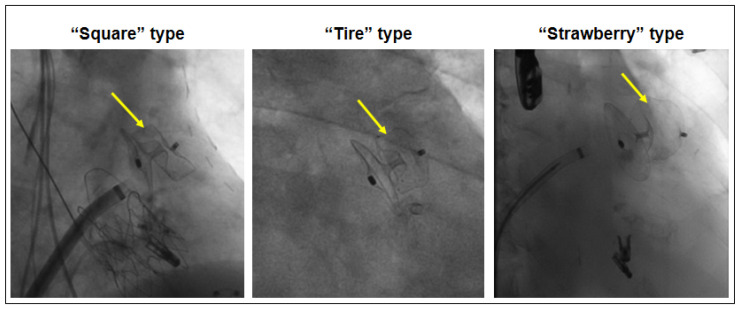
LAA occluder lobe device morphology (yellow arrows) after implantation by fluoroscopy. “Square” type as a sign of low deformation of the device lobe indicating undersizing, “tire” type as the optimal deformation of the lobe, and “strawberry” type as a sign of excessive deformation of the lobe indicating oversizing of the LAA occluder device.

Immediately after the LAA occluder implantation, the lobe morphology of the device was defined by fluoroscopy as “square” type (sign of low deformation of the device lobe), “tire” type (optimal deformation of the lobe ensuring secure fitting and achieving the best LAA sealing), and “strawberry” type (sign of excessive deformation of the lobe) [15] (Figure 2). The classification into these three categories was repeated by a second experienced physician for the first 30 patients at two different points in time to control for inter- as well as intra-rater variability. The detailed steps of the implantation technique are described elsewhere [14].

### 2.4. Statistical Analysis

Statistical analyses were performed using MedCalc statistical software Version 13.0 (MedCalc Software Ltd., Ostend, Belgium). The categorical variables were indicated as count (percentage). The continuous variables were expressed as mean (±standard deviation). The agreement of measurements with different imaging methods was compared with Student’s paired *t*-test. The occurrence of PDLs according to the postprocedural deformation type of the device lobe was analyzed by Pearson’s chi-square test. The eccentricity index of the LAA device landing zone was compared according to the deformation type of the device lobe by analysis of variance (ANOVA). A receiver-operating characteristic (ROC) curve analysis was performed to calculate the area under the curve (AUC) with the corresponding 95% confidence interval (95%-CI) for the prediction of “strawberry”-type deformation of the device lobe according to the eccentricity index of the device landing zone. The impact of the 3D TEE eccentricity index on the occurrence of postprocedural PDLs was calculated by an independent samples *t*-test. The agreement in the classification of the device lobe morphology as “square” type, “tire” type, and “strawberry” type between two different raters and the same rater at two different points in time was calculated for the first 30 patients using the Cohen’s kappa statistic.

*p*-values less than 0.05 were defined as statistically significant.

## 3. Results

In this single-center study, 47 consecutive patients (76 ± 8 years; 29 male) were retrospectively included. Table 1 shows the patients’ clinical and procedural characteristics.

The mean CHA_2_DS_2_-VASc score was 4.3 ± 1.3, and the mean HAS-BLED score was 3.6 ± 1.0, indicating a high risk for stroke and bleeding. In all the patients, the indication for LAA occlusion was a history of bleeding. LAA occlusion with an Amplatzer^TM^ device was successfully performed in all the patients: in 16 (34%) patients, an Amplatzer^TM^ ACP device was implanted, and in 31 (66%) patients, it was the Amplatzer^TM^ Amulet^TM^ device. Device-related thrombi occurred in three (6%) patients. Two thrombi (one already detected during intervention and one after eight months incidentally in a CT scan) were successfully treated by oral anticoagulation with Phenprocoumon. The third thrombus was detected after three months by TEE, and this patient was in need of cardiac surgery due to the unfortunate size and localization of the thrombus.

At the 6-month TEE follow-up, a PDL was present in 11 (23%) patients (PDL < 3 mm in 7 (15%), 3–5 mm in 4 (9%), and >5 mm in 0 (0%) patients) (Table 2).

### 3.1. LAA Sizing

Table 3 shows the measurements of the device landing zone by CCA in two different projections: by 2D TEE with three different views and by 3D TEE with maximal and minimal diameter, area-derived mean diameter, and ratio of the maximal and minimal diameters by 3D assessment.

The device size was selected depending on the measurements of the maximal diameters by CCA: the maximal diameter of the device landing zone by CCA was 22.2 ± 3.9 mm and was comparable to the maximal diameter by 3D TEE with 22.2 ± 4.1 mm (*p* = 0.843), whereas 2D TEE showed smaller maximal diameters with 19.7 ± 3.7 mm (CCA vs. 2D TTE, *p* < 0.001; 3D TEE vs. 2D TEE, *p* < 0.001).

### 3.2. Device Morphology After Implantation and Peri-Device Leak

In 28 (60%) patients, a “tire”-type morphology as the optimal deformation of the lobe was documented; in 9 (19%) patients, a “square”-type morphology and, in 10 (21%) patients, a “strawberry”-type morphology was present. The inter-rater and intra-rater agreement in the classification of the morphology of the device lobe as “square” type, “tire” type, and “strawberry” type was very good (weighted Kappa 0.803, 95%-CI 0.620 to 0.986, and 0.848, 95%-CI 0.683 to 1.000, respectively) indicating reliable reproducibility of grouping into these categories.

“Tire”-type morphology was associated less frequently with PDLs (11%) than “square” type (56%) and “strawberry” type (30%) (*p* = 0.019) (Table 4, upper row).

The eccentricity of the device landing zone was assessed by preprocedural 3D TEE and calculated as the ratio of the maximal and minimal diameters. Patients with postprocedural “strawberry”-type lobe deformation of the device tended to have a more eccentric shape of the device landing zone (eccentricity index: ratio max.-min. diameter = 1.45) than patients with “square”-type or “tire”-type morphology that had a more circular shape (eccentricity index: ratio max.-min. diameter = 1.35, *p* = 0.306). The eccentricity index of the landing zone of the Amplatzer^TM^ device assessed by 3D TEE tended to be higher in patients with postprocedural PDLs (1.40 ± 0.34) than that in patients without PDLs (1.37 ± 0.24; *p* = 0.135).

The 3D TEE eccentricity index with a cut-off value of ≤1.29 was a very specific predictor for excluding postprocedural device deformation of the “strawberry” type (AUC = 0.689, 95%-CI 0.538 to 0.816; sensitivity 48.7% and specificity 100%), which results from oversizing due to inadequate device selection by CCA measurements in a more eccentric morphology of the landing zone (Table 4, lower row).

## 4. Discussion

The major findings of this study were as follows: (1) the maximal diameters of the LAA landing zone measured by 3D TEE were comparable to the measurements by CCA, and these measurements were larger than those by 2D TEE; (2) optimal deformation in fluoroscopy with a “tire”-type morphology of the Amplatzer^TM^ device lobe was associated with a lower occurrence of a postprocedural PDL at 6-month follow-up in contrast to “square”-type and “strawberry”-type morphology; (3) the preprocedural eccentricity of the device landing zone of the LAA assessed by 3D TEE as the ratio of the maximal and minimal diameters (eccentricity index) was a predictor for device oversizing indicating inadequate size selection of the Amplatzer^TM^ device.

### 4.1. LAA Measurements

Precise measurements of the LAA dimensions are important for the selection of the correct device size. Preprocedural TEE as the most frequently used method and cardiac CT are recommended to assess the maximal diameter of the LAA [6]. For correct selection of the device size of the LAA occluder, the Amplatzer^TM^ instructions for use recommends using two different imaging modalities, such as 2D TEE and CCA. Several studies have shown that 2D TEE diameters tend to be 2–3 mm smaller than those obtained by cardiac CT, which better identified the correct device size [7,8,9]. The measurements by CCA vary in different studies: Clemente et al. (2015) showed a good correlation between mean diameters measured by cardiac CT and the largest diameters obtained by CCA, which were both larger than the maximal diameters measured by 2D TEE, whereas Saw et al. (2016) reported the smallest diameters measured by CCA [9,16]. Complicating the sizing process, the LAA size can fluctuate depending on the cardiac rhythm and volume status; therefore, factors like fasting and fluid administration may alter the LAA dimensions obtained by various imaging modalities [6,17,18].

In our study, the smallest average maximal diameters of the device landing zone were measured by 2D TEE, which were 2.5 ± 3.2 mm smaller than those measured by CCA and 2.6 ± 3.4 mm smaller than those measured by 3D TEE. According to the instructions for use, this difference in device landing zone diameter would lead to smaller device size selection by 2D TEE alone. The average diameters of the device landing zone by CCA and 3D TEE were comparable (22.2 ± 3.9 mm and 22.2 ± 4.1 mm, *p* = 0.843), and therefore, 3D TEE showed better accuracy for device size selection than 2D TEE in our study. In another study, Wang et al. (2016) also reported larger diameters measured by 3D TEE in comparison to 2D TEE, but the 3D TEE diameters were smaller than those obtained by CT [7]. Previous studies have confirmed the accuracy of device sizing by 3D TEE [18,19], and the use of 3D TEE is increasingly endorsed since 3D TEE imaging improves the visualization of complex LAA morphology, is free of radiation and contrast medium in contrast to cardiac CT, and can be obtained during preprocedural and intraprocedural TEE [6,14].

### 4.2. Peri-Device Leak

The LAA anatomy is highly variable, and missed lobes as well as sub-optimal device deployment may cause PDLs [4,20]. PDLs of any size are common with an incidence of 37% for the Amplatzer^TM^ device at TEE follow-up, leading to an increased risk for stroke, systemic embolism, and cardiovascular death [21,22]. Therefore, complete sealing of the LAA is important.

The Amulet IDE trial showed that the Amplatzer^TM^ Amulet^TM^ device had fewer severe PDLs (>5 mm) than the WATCHMAN^TM^ 2.5 device. One risk factor for PDLs with the WATCHMAN^TM^ device was a large LAA dimension, whereas there were no anatomical predictors for PDLs with the Amulet^TM^ device [4]. Unlike the Amulet IDE trial, our study identified an anatomical predictor using 3D TEE: the preprocedural eccentricity index, defined as the ratio of the maximal and minimal 3D TEE diameters, was higher in the unfavorable “strawberry” morphology of the device lobe, indicating device oversizing and higher rates of PDLs than in the optimal postprocedural device morphology. Similarly, other studies in patients with LAAO with plug-type devices showed that preprocedural assessment of the eccentricity index by computed tomography identified a more eccentric LAA orifice morphology as a predictor for PDLs [10,11]. In contrast, Nirmalan et al. found no significant association between eccentricity and PDLs but rather identified malalignment of the device as the main contributor to this complication [12]. The impact of eccentricity on PDLs as assessed by CT therefore remains controversial. However, in more eccentric LAA landing zones, the LAA occluder size has to be chosen carefully, and oversizing by too large a device selection should be critically discussed. Accordingly, Lakkireddy et al. (2023) demonstrated that both oversizing and undersizing were predictors of severe PDLs [4]. In our study, intraprocedural device morphology by fluoroscopy was also associated with postprocedural PDLs at the 6-month follow-up: “tire”-type morphology of the Amplatzer^TM^ device as sign of optimal device deformation showed a lower occurrence of postprocedural PDLs at 6-month follow-up in contrast to device malformation with “square”-type and “strawberry”-type morphology.

Korsholm et al. (2021) published that, after Amplatzer^TM^ (ACP/Amulet^TM^) device implantation, intraprocedural TEE identified small PDLs (<3 mm) in 15% of all patients with an increase to 32% at the 8-week TEE follow-up [23]. Despite intraprocedural exclusion of PDLs, PDLs can evolve and deteriorate after LAA occluder implantation, and therefore, the evaluation of further predictors of later PDLs like intraprocedural device morphology by fluoroscopy seems reasonable for optimal procedural success.

### 4.3. Limitations

This study shows the data of a retrospective, monocentric, relatively small cohort with restricted evaluation of prognostic data. Cardiac CT is not routinely performed prior to LAA occlusion at our institution. Therefore, no cardiac CT data were collected for this study. Nevertheless, a comparison of CCA and TEE diameters with cardiac CT diameters would have been of interest as the study situation is partly contradictory. Since only LAAO with the Amplatzer^TM^ device was examined, these results cannot be extrapolated for other LAAO devices. Moreover, further studies are needed to evaluate whether the selection of device size considering the 3D TEE eccentricity index as the ratio of the maximal and minimal diameters of the LAA landing zone can reduce device mis-sizing and the occurrence of PDLs. Furthermore, a PDL has a lower detection rate by TEE than by CT [23]. Therefore, some cases of PDLs might have been missed when assessed by TEE alone.

In the case of a so-called LAA chicken-wing anatomy with a pronounced bend and a short LAA neck, a special implanting method called the “sandwich” technique may be applied. The main characteristic of this technique is that the device lobe is not implanted in the standard neck position but rather along the length of the LAA body. In our study, in 2 of the 47 patients, the “sandwich” implanting technique was used, in which a typically less pronounced lobe deformation is observed. These two patients had no strawberry deformation. The inclusion of these patients into the analysis may have influenced the results. However, our aim was to evaluate the preprocedural imaging independent of the implantating technique used for the Amplatzer^TM^ device. Moreover, LAA visibility at 0° was not sufficient in about 15% of patients so that relevant anatomical details could be missing, which may have affected the study’s results.

## 5. Conclusions

Undersizing and oversizing of the LAA occluder device as characterized by deformation type in fluoroscopy were associated with postprocedural PDLs. The 3D TEE eccentricity index of the LAA landing zone was a predictor of inadequate size selection of the Amplatzer^TM^ device resulting in oversizing in patients with a more eccentric LAA anatomy and correlating with the occurrence of PDLs. Therefore, 3D TEE may be used for better intraprocedural guidance and correct size selection of the Amplatzer^TM^ device.

## Figures and Tables

**Table 1:** Patient characteristics.

Variables	*n* = 47
Age, years	76 ± 8
Male gender, *n* (%)	29 (62)
Type of atrial fibrillation	
Paroxysmal, *n* (%)	21 (45)
Persistent, *n* (%)	8 (17)
Permanent, *n* (%)	18 (38)
CHA_2_DS_2_-VASc score	4.3 ± 1.3
HAS-BLED score	3.6 ± 1.0
NYHA class	
I, *n* (%)	21 (45)
II, *n* (%)	12 (26)
III, *n* (%)	12 (26)
IV, *n* (%)	2 (4)
Arterial hypertension, *n* (%)	32 (68)
Chronic kidney disease, *n* (%)	19 (40)
Congestive heart failure, *n* (%)	21 (45)
Prior stroke, *n* (%)	9 (19)
Prior bleeding, *n* (%)	47 (100)
Indication for LAA occlusion	
Intracranial bleeding, *n* (%)	11 (23)
Gastrointestinal bleeding, *n* (%)	20 (43)
Urogenital bleeding, *n* (%)	6 (13)
Other causes for bleeding, *n* (%)	10 (21)
Medication on admission	
DOAC/Phenprocoumon/Enoxaparin, *n* (%)	17 (36)

Values indicate mean ± standard deviation or (%) of all patients. CHA_2_DS_2_-VASc: congestive heart failure, hypertension, age ≥ 75 years, diabetes mellitus, prior stroke or transient ischaemic attack or thromboembolism, vascular disease, age 65–74 years, sex category, DOAC: direct oral anticoagulant, HAS-BLED: hypertension, abnormal renal/liver function, stroke, bleeding history or predisposition, labile INR, elderly, drugs/alcohol concomitantly, LAA: left atrial appendage, NYHA: New York Heart Association.

**Table 2:** Procedural characteristics and data.

Variables	*n* = 47
LAA occluder device	
Amplatzer^TM^ ACP device, *n* (%)	16 (34)
Amplatzer^TM^ Amulet^TM^ device, *n* (%)	31 (66)
Adverse events	
Periprocedural pericardial effusion, *n* (%)	3 (6)
Postprocedural device-related thrombus, *n* (%)	3 (6)
Peri-device leak at 6-month follow-up, mm	2.6 ± 0.7
PDL > 5 mm, *n* (%)	0 (0)
PDL 3–5 mm, *n* (%)	4 (9)
PDL < 3 mm, *n* (%)	7 (15)
Medication on discharge	
Phenprocoumon/Enoxaparin, *n* (%)	2 (4)
Aspirin and Clopidogrel, *n* (%)	45 (96)

Values indicate mean (±standard deviation) or % of all patients. ACP: Amplatzer^TM^ Cardiac Plug, LAA: left atrial appendage, PDL: peri-device leak.

**Table 3:** Measurements of the device landing zone by conventional cardiac angiography (CCA), 2D TEE, and 3D TEE.

	CCA	2D TEE	3D TEE
Diameter RAO 30° cranial 20° (mm)	21.0 ± 3.6	-	-
Diameter RAO 30° caudal 20° (mm)	21.4 ± 4.2	-	-
Diameter 45° (mm)	-	17.0 ± 3.4	-
Diameter 90° (mm)	-	17.4 ± 3.7	-
Diameter 135° (mm)	-	18.8 ± 3.8	-
Area by 3D assessment (mm^2^)	-	-	293.0 ± 113.9
Area-derived mean diameter by 3D assessment (mm)	-	-	19.0 ± 3.6
Maximal diameter (mm)	22.2 ± 3.9	19.7 ± 3.7 *	22.2 ± 4.1 ^#^
Minimal diameter (mm)	-	-	16.5 ± 3.5
Eccentricity index (ratio of maximal and minimal diameter by 3D assessment)	-	-	1.37 ± 0.26

CCA = conventional cardiac angiography, RAO = right anterior oblique, TEE = transesophageal echocardiography. * CCA vs. 2D TEE: *p* < 0.0001 and 3D TEE vs. 2D TEE: *p* < 0.0001. ^#^ CCA vs. 3D TEE: *p* = 0.843.

**Table 4:** Occurrence of peri-device leaks (PDLs) according to postprocedural deformation type of the LAA occluder device lobe and preprocedural eccentricity of the LAA device landing zone assessed by 3D TEE.

	“Square” Type (*n* = 9)	“Tire” Type (*n* = 28)	“Strawberry” Type (*n* = 10)
Peri-device leak after procedure	5 (56%)	3 (11%) *	3 (30%)
Ratio max.-min. diameter by 3D TEE	1.35 ± 0.28		1.45 ± 0.17 ^#^

LAA: left atrial appendage, TEE: transesophageal echocardiography. * “Tire” type vs. “Square” and “Strawberry” type, *p* = 0.019. **^#^** “Strawberry” type vs. “Square” and “Tire” type, *p* = 0.306.

## Data Availability

The data presented in this study are available on request from the corresponding author. The data are not publicly available due to patient privacy.
